# The Theory of Effort Minimization in Physical Activity

**DOI:** 10.1249/JES.0000000000000252

**Published:** 2021-04-08

**Authors:** Boris Cheval, Matthieu P. Boisgontier

**Affiliations:** 1 Swiss Center for Affective Sciences; 2 Laboratory for the Study of Emotion Elicitation and Expression (E3Lab), Department of Psychology, University of Geneva, Geneva, Switzerland; 3 School of Rehabilitation Sciences, Faculty of Health Sciences, University of Ottawa; 4 Bruyère Research Institute, Ottawa, ON, Canada

**Keywords:** automatic behavior, brain, exercise, health behavior, neuropsychology, psychology, sedentary behavior

## Abstract

Although the automatic attraction to effort minimization has been evidenced in multiple fields, its potential role in explaining the pandemic of physical inactivity has been overlooked. The theory of effort minimization in physical activity (TEMPA) fills this gap. TEMPA seeks to obtain a more accurate understanding of the neuropsychological determinants of movement-based behaviors.

## Abstract 2

We put forward a theoretical framework aiming to develop a more accurate understanding of the neuropsychological determinants of physical activity.

**Key Points**

- Many individuals intending to be physically active fail to turn these intentions into action.
- Automatic affective associations with physical activity could explain the gap between intentions and action.
- Although the automatic tendency for effort minimization widely has been evidenced in multiple fields, its role in the regulation of movement-based behaviors largely has been ignored.
- The theory of effort minimization in physical activity (TEMPA) integrates automatic reactions to physical activity cues and automatic attraction toward effort minimization into a single framework.
- TEMPA is designed to achieve a more complete and accurate understanding of the neuropsychological mechanisms involved in the self-regulation of movement-based behaviors.

## INTRODUCTION

Imagine you planned to go for a walk this morning, but you got stuck to your chair. What are the forces that could explain this failure to engage in physical activity? Is it a paucity of a driving force toward the behavior to be achieved (walking), too much resistance posed by the behavior to be avoided (sitting in your chair), or a combination of the two? The theory of effort minimization in physical activity (TEMPA) presented in this article intends to answer these questions. TEMPA provides a theoretical framework to explain why many individuals intending to be physically active fail to turn these intentions into action (1).

The involvement of automatic processes in the regulation of movement-based behaviors is accepted widely now (2). Numerous studies testing these automatic processes have shown that physical activity cues attract attention, trigger positive affective reactions, and produce approach tendencies, especially in the most active people (3–5). These results suggest that automatic responses to physical activity cues that are insufficiently positively valued can partly explain failures to engage in physical activity. However, a strong automatic attraction toward effort minimization could be another explanation. Neuroscientific studies testing decision making have shown that humans favor behaviors associated with lower effort (6–8). Yet, effort minimization largely has been ignored in studies investigating the determinants of human engagement in physical activity. Hence, the role of this automatic attraction toward minimizing effort in the current pandemic of physical inactivity remains unclear (9).

TEMPA offers a new perspective on the neuropsychological determinants of movement-based behaviors that integrates automatic reactions to physical activity cues and automatic attraction toward effort minimization in a single framework. This automatic attraction is discussed based on a neuropsychological approach to physical effort (6–8) anchored in an evolutionary perspective (10,11). In the first part of this article, we briefly describe the dual-process theories of health behaviors and their recent applications to physical activity. Afterward, we explain how humans have evolved to be physically active but in an efficient way, through favoring behaviors that minimize effort. Next, we present TEMPA. Finally, we discuss the implications of this new theoretical framework for basic and applied research investigating the determinants of movement-based behaviors such as physical activity and sedentary behaviors.

In this article, we consider human behavior on an *energetic continuum*, with *sedentary behaviors* referring to any waking behavior characterized by an energy expenditure of 1.5 metabolic equivalent of task (MET) or lower while sitting, reclining, or lying down (12), and *physical activity* as any waking behavior characterized by an energy expenditure superior to 1.5 MET produced by bodily movements (13). Of note, positioning sedentary and physical activity behaviors on an energetic continuum does not prevent these behaviors from having their own motivational antecedents and health consequences, which have been widely reported in the literature (14). *Movement-based behaviors* are the behaviors enacted for everything we do, including sitting, standing, and different intensities of physical activity (15). *Movement-related cues* are cues related to movement-based behaviors. *Exercise* is considered as a subcategory of physical activity that is planned, structured, repetitive, and aims to improve or maintain one or more components of physical fitness (13). *Physical inactivity* is not considered as a behavior but as a level of physical activity that is not sufficient to meet physical activity recommendations (12). *Energy* is considered as the ability to produce physical action. *Effort* is thought as the level of cortical activity associated with the initiation or maintenance of a behavior. The brain constructs this perception based not only on the current physical effort that is elicited but also on previous experience of similar effort, motivation, awareness, and affects (16). *Effort minimization* is defined as the process that aims to achieve the most cost-effective behavior based on this perception. This terminology is applied throughout the article except for names of previous theories.

## DUAL-PROCESS MODELS

### Dual-Process Models of Health-Related Behaviors

In recent years, new dual-process models of health-related behaviors including physical activity have been developed (10,17,18). These theoretical perspectives suggest that physical activity behaviors are governed not only by controlled processes (*e.g.*, attitudes, intentions) but also by automatic processes (*e.g.*, automatic affective reactions or approach-avoidance tendencies).

*Controlled processes*, also referred to as reflective processes, rely on higher brain functions. They are slow and deliberative, requiring cognitive resources and involving conscious awareness (19). In contrast, automatic processes rely on well-learned associations and heuristic cues. Automatic processes are faster and initiated unintentionally, taxing cognitive resources to a much lesser extent, and not requiring conscious awareness. For example, when exposed to a physical activity cue, people may automatically activate memories associated with the concept of physical activity, such as the positive affects experienced during a previous physical activity. This type of positive affective association is likely to increase the engagement in a behavior, whereas negative affective associations are likely to decrease it (19).

The lack of consideration of *automatic processes* could account for the difficulties of the dominant models of health-related behaviors, such as sociocognitive and control models (2), to explain failures in turning intentions into behaviors (1). According to dual-process models, both controlled and automatic processes should be considered to accurately predict behaviors. Automatic processes can facilitate the execution of the intended behavior. For example, when a person is motivated to be active and has developed positive affective associations with physical activity, both processes are convergent and support the execution of the same behavior: being physically active. However, controlled and automatic processes also can be discordant. This is the case when a person who intends to be physically active has developed negative affective associations with physical activity. In this situation, whether the active behavior will be implemented depends on the controlled resources of the individual, their availability, and the nature of the behavior to be implemented. These moderators need to be taken into account, as individuals with low self-control or cognitive resources experience more difficulties in implementing the active behavior (20–22). Yet, the availability of these resources also is dependent on various factors. Individuals who are mentally fatigued, under high-cognitive load, or exposed to stress experience more difficulties to recruit their controlled resources. Finally, the extent to which the intended behavior already has been automatized affects the amount of brain resources required to implement this behavior.

**Figure 1:**
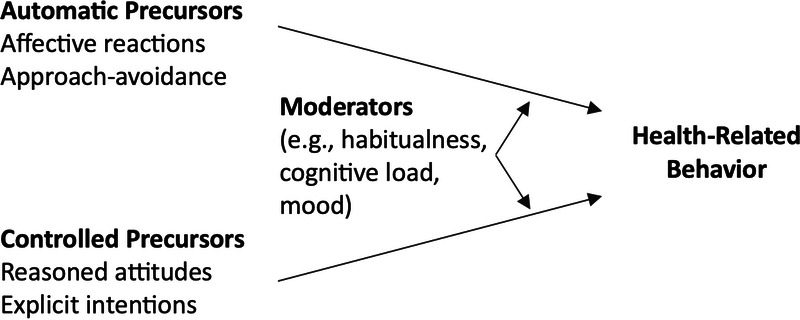
Dual-process framework for the prediction of health-related behaviors.

In summary, dual-process models suggest that a better understanding of the regulation of health-related behaviors requires the conceptualization of a system that would include controlled processes, automatic processes, and moderators that affect the strength of these processes (Fig. 1).

### Dual-Process Models of Physical Activity Behaviors

As observed in sociocognitive theories of physical activity (24), recent theoretical efforts have been performed to develop idiosyncratic dual-process models of physical activity (10,17,18). The vast majority of the literature assessing automatic processes in physical activity relies on general dual-process models, such as the reflective-impulsive model (19) or the associative-propositional evaluation model (23). Here, we briefly describe the conceptual dual-process model focusing on the automatic affective evaluation of physical activity (18) and the affective-reflective theory of physical inactivity and exercise (17). Afterward, we show that these two theories accurately model the predictive value of learned affective experiences associated with physical activity but do not incorporate boundary conditions that could mitigate these predictions, such as the automatic attraction toward effort minimization. By considering both the importance of these learned associations and the essential role of the automatic attraction toward effort minimization, TEMPA supplements the previous approaches. As such, TEMPA provides a more accurate understanding of the neuropsychological mechanisms underlying the self-regulation of movement-based behaviors.

### Automatic Affective Evaluations of Physical Activity

In their review anchored in prominent dual-process models (19,23), Conroy and Berry (18) suggest that automatic affective evaluations are instrumental to the regulation of physical activity. Specifically, they argue that the concept of physical activity can be paired with positive or negative affective reactions (pleasure and displeasure) due to the repeated experience of their concomitance. As a result of this learned association, contextual cues that trigger the concept of physical activity also trigger an *automatic affective evaluation of physical activity*. This automatic affective evaluation that occurs rapidly and effortlessly can shape subsequent controlled processes related to physical activity, such as attitudes and intentions. The automatic affective evaluation of physical activity is connected to an approach-avoidance impulse that increases or reduces the likelihood to engage in physical activity. In this framework, the displeasure experienced during physical activity explains the difficulty to engage in regular physical activity. As the automatic system is based on associations, this system is more likely to influence spontaneous, unplanned actions such as light-intensity physical activities. In contrast, the controlled system relies on rules and propositions and is therefore more likely to influence behaviors that require explicit monitoring and volitional actions, such as exercise behaviors.

### Affective-Reflective Theory of Physical Inactivity and Exercise

The affective-reflective theory (17) also is rooted in dual-process models (19,23) and suggests that external (*e.g.*, a friend's reminder that you had planned to go to the gym) and internal (*e.g.*, remembering that you intended to go to the gym) physical activity cues activate automatic associations resulting in an *automatic affective valuation* of these cues. The automatic affective valuation is defined as the unattended and tacit assignment of a positive value (*i.e.*, pleasure) or negative value (*i.e.*, displeasure) to a cue. This valuation is believed to result from affective perceptions repeatedly experienced during physical activity (*e.g.*, sense of physical reinvigoration vs bodily discomfort) or to be mediated by cognitive appraisals (*e.g.*, pride vs embarrassment) stemming from these repeated experiences. The automatic affective valuation precedes and can therefore affect controlled processes that rely on deliberative evaluations of physical activity. The automatic affective valuation of physical activity is connected to an impulse (*i.e.*, approach or avoidance) that prompts the individual to change the situation (*i.e.*, driving forces) or to stay in the situation (*i.e.*, restraining forces). In contrast, the controlled processes can result in an action plan. The impulse and the plan can be concordant (support the same response) or discrepant (support different responses). In the latter case, self-control resources determine whether automatic or controlled processes predominately guide the behavior. In brief, the affective-reflective theory regards the negative affective valuation of physical activity as a restraining force that may prevent individuals from implementing their reflective plans to be physically active; it posits that self-control resources are a critical moderator that can determine the behavior that will ultimately be performed (see also (25) for a discussion on this model).

### The Missing Automatic Affective Evaluation

The automatic affective evaluations of physical activity (18) and the affective-reflective theory (17) have multiple similarities. They both rely on two prominent dual-process models, the reflective-impulsive model (19) and the associative-propositional evaluation model (23). The terminology (*e.g.*, *automatic affective evaluation* vs *automatic affective valuation*) and the mechanisms underlying physical activity behavior (*e.g.*, automatic processes preceding and interacting with controlled processes) also closely match. In particular, the automatic processes included in these models fully rely on affective experiences associated with physical activity. These models argue that perceiving a cue related to physical activity automatically activates the concept of physical activity together with associated pleasant or unpleasant affective memories, which in turn leads to an impulse favoring the tendency to approach or avoid physical activity. This mechanism was first described by Williams and Evans (26), who argued that affect processing results from previous or anticipated affective responses to a health-related behavior. Overall, these models are consistent with the literature highlighting the importance of affective responses to exercise-related cues in the regulation of physical activity (27–29).

However, automatic processes related to affective experiences associated with a reduction, a cessation, or an absence of physical activity (*i.e.*, sedentary behaviors) were not considered despite experimental evidence demonstrating their involvement in the regulation of physical activity (3,7,22,30). In other words, the possibility that the concept of physical effort minimization can be paired with positive affective perceptions (pleasure) due to the experience of their repeated concomitance was not considered. Yet, the automatic affective evaluation of effort minimization cues resulting from this pairing is likely connected to an impulse that prompts an individual to change or maintain movement-based behaviors. Therefore, it seems essential to consider effort minimization in idiosyncratic models of physical activity.

In sum, by highlighting the important and hitherto overlooked role of the automatic affective evaluation of physical activity, the idiosyncratic dual-process models have advanced greatly the modeling of the neuropsychological processes underlying physical activity behaviors (17,18). Adding the automatic affective evaluation of physical effort minimization to this modeling likely would improve further its accuracy.

Physical effort can be associated with muscle fatigue, namely, the decrease in the ability to produce force, which may arise not only because of changes at the level of the muscle (peripheral fatigue) but also because the central nervous system fails to adequately drive the motoneurons (central fatigue) (31). In addition, physical effort often is processed as a negative experience to be avoided (8). Therefore, the repeated experience of its reduction contributes to a positive automatic affective evaluation of contextual cues related to its minimization. At least two pathways may help individuals avoid the implementation of behaviors minimizing physical effort in the presence of such contextual cues: i) a controlled pathway that relies on the elaboration of an action plan aiming to inhibit or compete with the automatic processes, and ii) an automatic pathway that relies either on the positive automatic evaluation of physical activity cues or on previously developed habits favoring energy consumption.

In the next section, we analyze the evolutionary origins of the automatic affective evaluation of movement-related cues. Based on evidence from multiple fields, we contend that humans have evolved to be physically active but in an efficient way, through favoring behaviors that minimize effort.

## EFFICIENT PHYSICAL ACTIVITY

### The Human Body and Its Functioning Are Shaped for Physical Activity

For over two million years, the anatomy and physiology of the human lineage have adapted to the high levels of physical activity required by the hunting strategy of our evolutionary ancestors. Specifically, bipedal hominin hunters combined endurance running and tracking to drive their prey into exhaustion or hyperthermia (32). This strategy of persistence was efficient because, due to sweating, the hunters had higher capacity to cool than their prey (32), but multiple adaptations further supported this efficiency. For example, whereas quadrupeds have a fixed 1:1 ratio of gait and breathing cycles, humans can decouple these cycles and optimize ventilatory efficiency. At a muscle level, humans exhibit a much higher ratio of slow-twitch fibers than chimpanzees. These fibers have high mitochondrial volume densities and capillary-fiber contact length, which facilitates O_2_ diffusion and improves endurance capacities. Human running efficiency has also been improved by the increased length of the triceps surae tendon, which is absent or short in great apes. The elastic recoil of this Achilles tendon can output 35% to 75% of the positive work required per stride (33). Similarly, the elastic properties of the longitudinal arch of the human foot that is absent in other primates can contribute 9% to 17% of the total limb mechanical work of running (34). Humans also developed features that enhanced stabilization during running, such as wider shoulders that increase the moment generated by upper-limb swinging, which counterbalance lower-limb swinging. Head stabilization has also been improved through the appearance of passive structures such as the nuchal ligament, which is absent in chimpanzees and australopithecines (35). These adaptations, together with a more extended hip and a longer hindlimb, also decreased cost of human walking, which is far more common than running among hunter-gatherers (36). Specifically, walking is 50% to 75% less costly than both quadrupedal and bipedal walking in chimpanzees (37,38). Finally, long-distance walking and running in hot environments became possible through the improvement of thermoregulatory capabilities, including the multiplication of eccrine sweat glands for evapotranspiration and reduced body hair that increases convection rates (35). All the aforementioned adaptations favored energetic efficiency and shaped humans as physically active living beings to the point that physical activity became essential to their health (9).

### Neuroendocrine Response to Physical Activity

Evolution has not only shaped the human body for long-distance running but has also conditioned the human brain to enjoy this type of physical activity through the development of hypoalgesic and mood-elevating neuroendocrine mechanisms. For example, physical activity has a hypoalgesic effect through the activation of the endogenous opioid and endocannabinoid systems (39). The hormones released by these systems also activate frontolimbic brain areas that are involved in the processing of affective states and contribute to the positive evaluation of endurance physical activity (40). The euphoric state resulting from these neuroendocrine mechanisms, referred to as the “runner's high” (40), increases humans' motivation to sustain high physical activity intensities over long distances and sometimes can become addictive (41). Genetics could also partly explain this runner's high (42). This biological explanation suggests that physical activity is tightly paired with hypoalgesic and mood-elevating mechanisms, which may result from their repeated concomitance across evolution. As a result of this pairing, the automatic affective evaluation of cues related to physical activity should be positive (*i.e.*, pleasurable). Automatic processes would, therefore, support any human intending to be physically active. Yet, the difficulty of engaging in a physically active lifestyle that many humans experience worldwide suggests that physical activity cues are not always evaluated positively.

Although the neuroendocrine response does improve the pleasure perceived during physical activities of low-to-moderate intensities, displeasure is the dominant perception during higher intensities (43). This displeasure extends to the postexercise affective response and often outweighs the typical neuroendocrine-related positive affective rebound experienced after exercise (43,44). This dependence on physical activity intensity could partly be explained by an inverted U relations with the hormonal release of endocannabinoids (45). Finally, although results suggest that the neuroendocrine response is absent after 30 min of walking (45), more studies are required to assess the effect of long-distance walking.

### Anaerobic Physical Activity

Ekkekakis *et al.* (43) argued that high-intensity physical activities relying on anaerobic metabolism could be responsible for the public health problem of physical inactivity. This suggestion is based on studies showing that when ventilation starts to increase (*i.e.*, ventilatory threshold), which reflects a transition from aerobic to anaerobic metabolism, most individuals report reduced pleasure and increased displeasure (43). This affective response could be explained by interoceptive adaptations that accompany the metabolic transitition. For example, adrenaline levels, which are linked to psychological stress, can be multiplied up to 15-fold during a 1.5-min anaerobic physical activity but are multiplied only 2- to 3-fold during a 50-min aerobic physical activity (46). Likewise, growth hormone levels, which improve well-being (47), decrease or do not change during anaerobic physical activity but can be multiplied 14-fold during aerobic physical activity (46). Fatigue and discomfort resulting from the accumulation of inorganic phosphate, which interferes with muscle activation processes (48), can also contribute to the reduced pleasure associated with anaerobic physical activity. Importantly, although these interoceptive adaptions likely are meant to keep the bodily systems within homeostatic conditions, they do not seem to affect the positive automatic affective evaluation of cues related to physical activity. Indeed, automatic reactions to theses cues, such as attentional capture (5), affective reactions (4), and approach tendencies (3), suggest that the evaluation of physical activity consistently favors physical activity behaviors, irrespective of the participants' usual level of physical activity. Therefore, the displeasure associated with anaerobic physical activity could not fully explain the pandemic of physical inactivity. Another explanation could be the positive automatic evaluation of cues that compete with the physical activity cues, such as the ones related to sedentary behaviors.

### Humans Have Evolved to Minimize Effort

Thus far, theories have suggested that high rates of physical inactivity in the general population could be explained by an evolved human tendency to avoid physical activities that are unnecessary (11). This innate tendency is thought to have developed through natural selection because it allowed the allocation of maximum energetic resources to reproductive activity and somatic maintenance (49,50). Consequently, cues related to sedentary behaviors would be evaluated positively (51), which would contribute to explain the physical inactivity pandemic (52). However, the discrete nature of this approach dichotomizing movement-based behaviors in physical activity on the one hand and sedentary behaviors on the other hand is limiting and prevents an accurate theorization of movement-based behaviors (10). As supported by the physical and neuroendocrinal adaptations described above, humans have evolved to minimize physical effort throughout the entire energetic continuum. Therefore, we argue that humans constantly seek efficient behaviors through multiple mechanisms, including the positive automatic evaluation of cues related to effort minimization.

Two mechanisms are involved in effort minimization: economy and efficiency. Economy can be defined as the reduction in energy expenditure, whereas efficiency is the ratio between the behavior accomplished and the energy expended, which indicates how well energy is converted into a useful or rewarding behavior. In other words, higher economy refers to lower energy expenditure, whereas higher efficiency refers to lower energy waste. The impact that efficiency can have on human behavior depends on the number of behavioral options that are available and the cognitive abilities required to make strategic decisions. For example, if the only possibility to reach a goal is to run, the only relevant boundary condition is economy, that is, how slowly should I run to preserve as much energy as possible but still reach the goal. If, in addition to running, the goal can also be reached using a bike, car, bus, or a combination of the former, efficiency comes into play, and higher reflective processes are required to make a strategic decision where not only economy but also energy waste and gain (*e.g.*, energetically denser food) are relevant factors. Across evolution, the development of new tools and technologies, together with the development of higher reflective abilities, has contributed to exponentially diversify the options for humans to interact with their environment. As a result, although economy was an essential behavioral determinant of our ancestors and remains at work in us, the weight of efficiency has been increasing over centuries to become prominent in modern societies.

This human tendency to minimize physical effort has been widely demonstrated in multiple fields, such as biomechanics (53), neuroscience (8), and evolutionary biology (54). For example, humans continuously optimize energetic costs during walking, including the modulation of walking speed, arm swinging, and step length, width, and frequency (53). In addition, studies have demonstrated that the energetic cost of movement drives motor adaptation during learning (55). Moreover, findings robustly confirmed that humans process physical effort as a cost in decision-making tasks and minimize the physical effort required to obtain a specific reward (6–8). Finally, anthropological data showed long periods of nonambulatory time in hunter-gatherers, thus suggesting that humans evolved in a context that included substantial inactivity (56).

In short, humans have evolved to be physically active but, more importantly, physically efficient. TEMPA integrates the processes underlying these opposite forces acting on human movement–based behaviors in a single framework.

## THE TEMPA

### Permanent Automatic Attraction to Effort Minimization

In TEMPA, the automatic attraction to physical effort minimization is conceptualized as a neuropsychological process at the level of the individual favoring the implementation and development of cost-effective behaviors. TEMPA posits that movement-related cues are perceived as effortful and that this effort is processed as a cost, that is, an aversive object to be avoided or minimized. Any movement, including breathing, constitutes an effort-related cue. Therefore, effort minimization processes are active at every moment of the lifespan. Although the intensity of this attraction to effort minimization is never null, it varies as a function of the characteristics of the individual, behavior, and environment at a given moment. In other words, effort minimization is a permanent and dynamic constraint that influences multiple stages of the regulation of movement-based behaviors. When a behavior is instigated, a movement-related cue can trigger automatic and controlled evaluations supporting the engagement in physically active behaviors. Concurrently, the perceived effort associated with this potential engagement is evaluated as a cost. Accordingly, this movement-related cue activates processes that result in opposite automatic and controlled precursors of behavior that will influence the behavioral decision. Once the behavioral decision has been made, the motor plan specifying the organization of the movements constituting the behavior is sent to the muscles to execute the behavior. At that stage, effort minimization processes contribute to the efficiency of motor planning through a feedback loop carrying information related to the actual effort associated with the behavior. Although effort minimization is thought to play a key role in the regulation of movement-based behaviors, other factors such as environmental, time, and pain-related constraints also are involved (57,58). For example, the effect of effort minimization that favors the engagement in behaviors associated with lower physical effort (*i.e.*, taking the elevator) can be overridden to adapt to a time constraint (*i.e.*, slowing down the speed at which the elevator doors close), which would result in a behavior associated with higher effort (*i.e.*, taking the stairs) (58).

### Energy Intake, Energy Expenditure, and Body Weight

Food availability and physical activity are thought to be part of the same cycle, where alternating periods of food scarcity and abundance are associated with higher and lower physical activity, respectively (59). The increased physical activity during food scarcity is interpreted as a foraging behavior aiming at restoring energy reserves for reproduction and survival. In modern societies, this automatic trigger of physical activity no longer exists. The disappearance of this trigger has likely contributed to increase sedentary behavior (60), which has been associated with higher total energy intake (61). In other words, the lack of food scarcity has reduced energy expenditure and increased energy intake, thereby contributing to the increased worldwide prevalence of overweight and obesity (62). At the level of an individual, higher body weight resulting from higher energy intake increases the perception of effort associated with a given behavior, thereby decreasing the likelihood of engaging in this behavior and expanding energy. To sum up, food availability has become a constant in the equation but has been replaced by a new variable affecting movement-based behaviors: being overweight.

The processes underlying energy conservation are at work not only in periods of food scarcity but also to restore energy reserves when food is available. This energy restoration was vital for our ancestors to survive the next period of energy scarcity. In current obesogenic environments, where energy-dense food has become abundant (63), food scarcity may seem to be nonexistent, but on an evolutionary scale, it is not. Although modern societies have not experienced food shortage on a large scale for multiple decades, such a period is not long enough for our brain to evolve mechanisms to increase the engagement in physical activity solely for the purpose of energy expenditure (27).

Although food often is abundant in our societies, self-imposed reduction in energy intake is common during periods of dieting. After a diet, weight regain is typical, and at least one third of dieters regain more weight than they lost (64), which further demonstrates the tendency of the human body to conserve energy, even when there is no food shortage. In periods where the level of energy intake is lower than usual, such as during a diet, we posit that the perception of physical effort is lowered because the automatic attraction toward physical activity inherited from times of food scarcity opposes the automatic attraction toward effort minimization. The same reasoning stands for times of the day when individuals are hungrier compared with other times. In sum, human behavior seems to oscillate between times of low energy intake supporting the engagement in efficient energy expenditure to find food and times of higher energy intake meant to compensate or overcompensate for this expenditure and anticipate the next expenditure. This behavioral oscillation takes place on different time scales ranging from years to hours. Yet, in modern societies, the exposure to energy-dense food may have disrupted these oscillations, especially in individuals with a low socioeconomic status, which has been associated with higher exposure to energy-dense food (65) and higher consumption of this type of food (66), as well as lower levels of physical activity (67).

### Effects of Effort Perception on the Preparation, Initiation, and Continuation/Repetition of Behavior

Although previous literature investigating perceived effort mainly focused on the ability to sustain effort (68), TEMPA contends that expected effort also is essential to the preparation of a behavior and to the decision to initiate it. Specifically, low expected effort is thought to trigger automatic processes supporting the preparation of a response, without necessarily triggering its initiation. If the prepared response (*i.e.*, automatic precursors) is replaced by a goal-directed alternative before the prepared response can be initiated, the automatic precursor does not evolve to an overt behavior. For example, when walking, the vision of an elevator is associated with low expected effort, which triggers automatic processes supporting the preparation of gait reorientation toward the elevator. However, this behavior can be overridden if the individual decides to search for the stairs to accomplish his or her goal of being physically active. Individuals may also have developed habitual behaviors that spontaneously favor the stairs, which would save cognitive resources. Conversely, high expected effort is thought to inhibit the preparation of a response associated with the initiation of behavior that is perceived as effortful. After behavior initiation, the attraction toward effort minimization tends to minimize energy spent in the ongoing behavior. This effort minimization can be opposed by different processes including the motivation to sustain the behavior, stimuli that distract attention from the perception of effort (69), and the manipulation of effort perception (70,71). These processes reduce the perception of effort during the behavioral performance and could in turn decrease future expectations of effort associated with the behavior, thereby promoting its repetition and adherence to rehabilitation programs (69). In sum, the perception of effort can affect the continuation of an ongoing behavior and its repetition across time, as well as its preparation and initiation.

### Individual Differences in Physical Effort Attraction and Tolerance

Although TEMPA supports an overall attraction toward effort minimization, this framework also expects individual differences in the attraction of physical effort and tolerance, because such differences have been observed for cognitive effort (72). Specifically, results showed that some humans have low need for cognition and avoid cognitive effort, whereas others have high need for cognition and seek out cognitive effort. Although scales such as the rate of perceived exertion and the category-ratio scale have been developed to assess the perception of physical effort, there is no tool assessing how people value physical effort. Yet, a scale measuring the tendency to enjoy, or dislike, effortful physical activity would significantly contribute to improving our understanding of the perception of physical effort and its influence on behavioral decisions.

Although TEMPA is not primarily designed to understand movement-based behaviors in specific conditions where health is impaired, the inclusion of perceived effort in this theory may account for observations made in patients and contributes to a better understanding of physical inactivity in these patients. For example, the automatic negative evaluation of physical activity behaviors may be particularly pronounced in patients with chronic conditions (*e.g.*, obesity, rheumatoid arthritis or cardiovascular disease) who likely have experienced pain and discomfort during physical activity. Moreover, TEMPA could be used to investigate a variety of disorders in which motivation is deficient (*e.g.*, behavioral apathy) or uncontrolled (*e.g.*, hyperactive behaviors), and that may reflect an inability to accurately perceive physical effort or an altered sensitivity to this effort (73). Likewise, this dysregulation could be hypothesized in patients with anorexia nervosa, who are characterized by a physical hyperactivity (74).

Although TEMPA primarily aims to explain why some individuals may fail to turn their intentions into action, this theory can also explain why other individuals succeed at implementing them. Specifically, this success is thought to result from strongly controlled (*e.g.*, strong motivation) or automatic processes (*e.g.*, positive affective response to exercise) that overweigh the automatic processes supporting effort minimization. If the engagement in physical activity becomes excessive, the neuroendocrine response involved in the perception of physical effort may be dysregulated. Such dysregulation potentially could be evidenced in addiction to exercise (41), which is characterized by a loss of control leading to compulsive and excessive physical activity with symptoms similar to other addictions (41). For example, people with exercise addiction continuously increase the amount of exercise to achieve the same desired sense of euphoria (*i.e.*, habituation), experience withdrawal symptoms when they are forced to suddenly reduce or stop exercise (*e.g.*, irritability, anxiety, fatigue, depression), and report that the excessive engagement in physical activities interferes with the quality of their familial, social, and professional lives (75).

### Physiological State

TEMPA places importance on the individual's physiological state during movement-related cue exposure, as this state influences the automatic evaluation of the cues. For example, when fatigued, humans may perceive that a behavior requires more effort than when they are not fatigued (76). As a result, the same physical activity cues can be automatically evaluated differently as a function of the state of fatigue. Likewise, physical fitness, as derived from variables such as maximum oxygen uptake (V̇O_2_ max) or maximum muscle force, is expected to influence the automatic evaluation of movement-related cues (*e.g.*, pleasure vs displeasure associated with running at a moderate pace). The physiological state also is likely to affect the perception of effort, which should be lower in less fatigued or fitter humans, thereby reducing the perceived necessity to decrease effort.

These suggestions are in line with previous findings showing that the perception of a stimulus is dependent on the physiological state of an individual. For example, hungry humans show stronger automatic approach tendencies toward food-related cues (77). It follows that fatigued and unfit humans may show stronger attraction to effort minimization cues. In addition to increasing fatigue, recent physical activity can also devaluate the potential rewarding value associated with physical activity (*i.e.*, outcome devaluation), as the need for physical activity has just been fulfilled, thereby further reinforcing the attraction to effort minimization cues.

In other words, TEMPA includes the moderating effect of the physiological state on the automatic evaluation of movement-related cues. This moderating effect accounts for the dynamic nature of automatic evaluations of movement-related cues in the short (*e.g.*, over 1 d) and long term (18). TEMPA also accounts for the effect of conditions affecting human fatigability and fitness, such as obesity, aging, and chronic disease.

### Environment

Besides the individual factors discussed previously, TEMPA also considers the effect of environmental factors on the regulation of movement-based behaviors because they constitute the external cues triggering the automatic and controlled processes at the root of these behaviors. These external cues can be related to movement and directly affect movement-based behaviors. Other cues can be unrelated to movement and contribute to the noise surrounding the signal carried by movement-related cues, thereby indirectly affecting movement-based behaviors. Movement-related cues depend on a broad range of factors related mainly to town planning, such as sidewalks, bikeways, and parks (78). In line with this suggestion, several studies have shown associations between characteristics of the physical environment and physical activity levels (79). Perceived safety and aesthetic features of the environment have also been associated with levels of physical activity, although less consistently. In other words, individuals surrounded by safe and appealing facilities or public spaces related to physical activity are more likely to be exposed to physically active cues and to positively evaluate these cues than individuals living in a neighborhood without such facilities and spaces.

TEMPA also posits that the activation of controlled or automatic processes by movement-related cues partly depends on the type of physical activity these cues are related to. For example, cues related to the physical activities of daily life are expected to rely mainly on automatic processes, whereas exercise-related cues are expected to rely mainly on controlled processes. Consistent with previous literature (11), TEMPA also suggests that cues related to emergency, play, or necessity (*e.g.*, fulfilling a need such as eating, foraging, or reproduction) reduce the perceived effort and its effect on controlled and automatic processes. This feature of emergency cues is meant to allow the rapid activation of a fight-or-flight response to protect the individual from an imminent threat or danger.

In sum, as suggested by the sociological framework (2), movement-based behaviors are influenced by complex interactions between environmental and individual factors. As such, environmental factors may either facilitate or hinder physical activity, but this effect is dependent on affects and motivation toward physical activity.

### Overview of TEMPA

**Figure 2:**
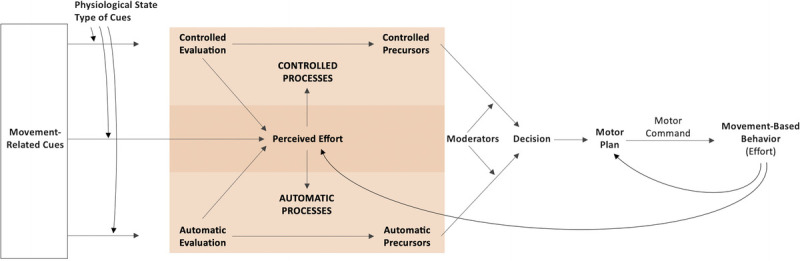
Theory of effort minimization in physical activity (TEMPA) framework for the prediction of movement-based behaviors. Movement-based behaviors are the behaviors enacted for everything we do and include sitting, standing, and different intensities of physical activity. Movement-related cues are cues related to movement-based behaviors. Controlled and automatic processes are defined as the mechanisms by which a person's behavior is regulated, whereas controlled and automatic precursors are the outputs of the processes (*e.g.*, intention and approach-avoidance tendencies). In TEMPA, movement-based behaviors are considered on an energetic continuum and depend on controlled and automatic processes that can be activated by internal and external movement-related cues. The positive or negative evaluation of these cues is dependent on the physiological state of the individual at the moment of exposure to these cues and on whether these cues are of a dispensable or necessary nature. The evaluation of the effort associated with the cues is affected by the positive or negative evaluation of these cues and will in turn influence the controlled and automatic processes leading to behavioral precursors. For these precursors to support the engagement in behaviors associated with an increased energy expenditure, the automatic and controlled processes supporting this engagement should be stronger than the processes supporting the minimization of the perceived effort. The relative weight of the controlled precursors (*e.g.*, reasoned attitudes, explicit intentions) and automatic precursors (*e.g.*, affective reactions, approach-avoidance tendencies) in the decision-making process is moderated by multiple factors (*e.g.*, habitualness, fatigue, cognitive load). The behavioral decision transforms the dominant behavioral precursor into an overt behavior through the implementation of a motor plan specifying the spatiality and temporality of the movements constituting the behavior that is sent to the muscles (*i.e.*, motor command). The resulting movement-based behavior requires a physical effort that will influence future perceptions and will be used in a feedback loop to update the motor plan and make it more efficient.

In TEMPA (Fig. 2), movement-based behaviors are considered on an energetic continuum and depend on controlled and automatic processes that can be activated by internal or external movement-related cues. The positive or negative evaluation of these cues is dependent on the physiological state of the individual at the moment of exposure to these cues and on whether these cues are of a dispensable or necessary nature. An essential innovation of TEMPA is the integration of perceived effort, which seems essential for an accurate model of movement-based behaviors. The evaluation of the effort associated with the cues is influenced by the positive or negative evaluation of these cues and will in turn influence the controlled and automatic processes leading to behavioral precursors (*e.g.*, intentions, approach-avoidance tendencies). For these precursors to support the engagement in behaviors associated with an increased energy expenditure, the automatic and controlled processes supporting this engagement should be stronger than the processes supporting the minimization of the perceived effort. The relative weight of the controlled precursors (*e.g.*, reasoned attitudes, explicit intentions) and automatic precursors (*e.g.*, affective reactions, approach-avoidance tendencies) in the decision-making process is moderated by multiple factors (*e.g.*, habitualness, fatigue, cognitive load). In individuals who have the intention to be physically active, controlled neuropsychological resources (*e.g.*, self-control) are expected to help overcome negative automatic evaluation of physical effort and favor physical activity engagement. However, when these controlled resources are lacking (*e.g.*, due to fatigue), the influence of automatic processes, which includes the automatic attraction to effort minimization, is increased. The behavioral decision transforms the dominant behavioral precursor in an overt action through the implementation of a motor plan specifying the spatiality and temporality of the movements constituting the behavior that is sent to the muscles (*i.e.*, motor command). The resulting movement-based behavior requires a physical effort that will influence future perceptions and will be used in a feedback loop to update the motor plan and make it more efficient.

Some predecisional processes are not explicitly included in Figure 2. For example, belief-attitude theories, competence-based theories, or control-based theories can be considered to explain behavioral intention. Moreover, postdecisional processes included in hybrid models that favor the successful translation of intention into behavior also are not illustrated. However, TEMPA is in line with sociocognitive theories that have been developed to address the intention-behavior gap, such as the action control frameworks, which suggests that self-regulatory constructs (*e.g.*, action planning) can explain the intention-behavior discordance (2,24,25).

## IMPLICATIONS FOR FUNDAMENTAL AND APPLIED RESEARCH

In this section, we briefly describe the implications arising from TEMPA for both fundamental and applied research. This section is not intended to be exhaustive, but rather to provide some examples that we consider promising.

### Controlling for Dispositional and Situational Factors

TEMPA suggests that the influence of perceived effort on the decision-making processes depends on dispositional (*e.g.*, physical fitness) and situational factors (*e.g.*, exercise history over the days or hours preceding the measurement). Therefore, the automatic evaluation of cues related to effort minimization, and their influence on decision-making, depends on factors such as maximum aerobic capacity, maximum muscle force, and recent exercise history. Yet, studies rarely control for the physiological state of the participants before and during the experiment (10). This absence of a control is an issue because in other contexts, the value that individuals assign to contextual cues clearly is influenced by their physiological state (77,80). Therefore, future studies should control for, and eventually manipulate, the effect of situational and dispositional factors on the processes driving movement-based behaviors. A corollary of the effect of situational factors is the effect of time. For example, the positive evaluation of cues related to effort minimization should strengthen over the course of physically active behaviors because the perceived effort increases due to fatigue. Time of day also could affect the strength of the automatic attraction toward effort minimization, as could the availability of cognitive resources to counteract this attraction. For example, the attraction can be higher when people are hungry and when cognitive resources are weak (*e.g.*, at the end of a working day). Testing the influence of time on the effect of cues related to effort minimization would be a way to demonstrate the dynamic nature of the automatic attraction toward this minimization.

### Reducing the Effect of Effort Minimization on Behavior

According to TEMPA, the automatic attraction to effort minimization is present throughout the energetic continuum. This suggestion accounts for the automatic adaptations reducing effort during physical activities, such as coordinating arm movements or adjusting step length (53). Although TEMPA posits that brain processes supporting effort minimization are permanently at work, this theory also argues that these processes can be influenced by physical and psychological factors. Several strategies could be considered to reduce the effect of effort minimization on behavior. One of these strategies is to reduce the sensitivity or increase the tolerance to physical effort. For example, a study showed that serotonin improves the ability to overcome the cost of effort (81). Specifically, the task involved trading handgrip force production against monetary benefits. Results showed that participants taking serotonin produced more force due to a diminished cost of effort. Nonpharmacological interventions aiming at improving physical fitness have also been shown to reduce effort cost (7). Removing or reducing the attention allocated to physical effort using distractive stimuli is another strategy that could be used to reduce the effects of effort minimization. For example, results showed that adding visual and auditory cues during a handgrip-squeezing task increased task adherence and was associated with lower levels of perceived effort compared with control conditions (occluded vision and no music) (82). Adding external stimuli is thought to divert attention from internal stimuli, thereby reducing the perception of effort and improving the affective experiences during exercise. A third strategy would be to manipulate psychophysiological feedback to bias the perception of effort (70).

Experiments investigating whether and how variations in effort perception can affect the automatic processes underlying the engagement in physical activity are needed still. These experiments could rely on immersive exercise tasks using virtual reality to manipulate the automatic processes associated with physical effort. For example, virtual reality could be used to create specific environments to associate effort with positive affective experiences (*e.g.*, showing beautiful landscapes during the physically active task). Finally, given the paucity of neuroscientific studies on the psychology of movement-based behaviors, studies investigating the neural structures and functions underlying the changes in physical effort integration should be encouraged.

### Altering Environment to Shape Behavior

TEMPA argues that humans have a spontaneous drive to minimize effort whenever the opportunity arises. Accordingly, environmental factors are thought to play a key role in shaping behaviors. Central to understating movement-based behaviors is taking into account the ubiquitous presence of low-effort opportunities in our environment and the automatic attraction toward them. Overlooking this variable would amount to investigating the difficulty to follow a diet without considering the availability of fatty and sugary food. As a result, promoting physical activity requires the development of an environment that triggers a spontaneous engagement in behaviors associated with higher rather than lower energy expenditure. To reach this goal, public policies can act on the availability and attractiveness of the opportunities to be less or more active. Regarding availability, a study showed that reducing the number of available escalators can improve the likelihood to take the stairs (83). Regarding attractiveness, developing innovative infrastructures such as staircases that play music could make the active behavior an enjoyable experience. Opportunities to expend less energy also could be made less appealing. For example, elevators are less appealing when door-closing time is longer (58). Reducing the visibility of elevators, making the stairs more aesthetically pleasing, providing easy access to space and equipment dedicated to active behaviors, and offering standing desks are other possibilities to make the environment more effort friendly. Yet, evidence supporting the effect of these interventions is still scarce.

## CONCLUSION

This article has discussed three key phenomena: the attraction to effort minimization, the cognitive resources allocated to resisting that attraction to effort minimization, and the affects involved in physical activity. Characterizing the interaction between these three factors, as well as the relative impact and power of each factor within that interaction, is necessary for understanding the gap between the intention to be physically active and actual outcomes. TEMPA is a theoretical framework that conceptualizes these interactions for constructing hypotheses and designing experimental studies aimed at solving the problem of physical inactivity. The overarching goal is to achieve a more complete and accurate understanding of the neuropsychological mechanisms involved in the self-regulation of movement-based behaviors.
